# Integrated analysis of colorectal cancer microRNA datasets: identification of microRNAs associated with tumor development

**DOI:** 10.18632/aging.101444

**Published:** 2018-05-18

**Authors:** Luca Falzone, Letizia Scola, Antonino Zanghì, Antonio Biondi, Antonio Di Cataldo, Massimo Libra, Saverio Candido

**Affiliations:** 1 Department of Biomedical and Biotechnological Sciences, University of Catania, Catania 95123, Italy; 2 Department of Pathobiology and Medical Biotechnologies, University of Palermo, Palermo 90127, Italy; 3 Department of Medical and Surgical Sciences and Advanced Technology "G.F. Ingrassia", University of Catania, Catania 95125, Italy; 4 Department of General Surgery, Vittorio Emanuele Hospital, University of Catania, Catania 95124, Italy; 5 Research Center for Prevention, Diagnosis and Treatment of Cancer (PreDiCT), University of Catania, Catania 95123, Italy; * Equal contribution

**Keywords:** colorectal cancer, microRNA, bioinformatics, dataset, biomarker

## Abstract

Colorectal cancer (CRC) is one of the leading cause of cancer death worldwide. Currently, no effective early diagnostic biomarkers are available for colorectal carcinoma. Therefore, there is a need to discover new molecules able to identify pre-cancerous lesions. Recently, microRNAs (miRNAs) have been associated with the onset of specific pathologies, thus the identification of miRNAs associated to colorectal cancer may be used to detect this pathology at early stages. On these bases, the expression levels of miRNAs were analyzed to compare the miRNAs expression levels of colorectal cancer samples and normal tissues in several miRNA datasets. This analysis revealed a group of 19 differentially expressed miRNAs. To establish the interaction between miRNAs and the most altered genes in CRC, the mirDIP gene target analysis was performed in such group of 19 differentially expressed miRNAs. To recognize miRNAs able to activate or inhibit genes and pathways involved in colorectal cancer development DIANA-mirPath prediction analysis was applied. Overall, these analyses showed that the up-regulated hsa-miR-183-5p and hsa-miR-21-5p, and the down-regulated hsa-miR-195-5p and hsa-miR-497-5p were directly related to colorectal cancer through the interaction with the Mismatch Repair pathway and Wnt, RAS, MAPK, PI3K, TGF-β and p53 signaling pathways involved in cancer development.

## Introduction

Colorectal carcinoma (CRC) is the most frequent tumor affecting the entire digestive tract. According to the data reported by GLOBOCAN 2012, CRC is the second most frequent cancer in women and the third most frequent in males. Overall, it is the fourth most frequent cancer in both sexes preceded only by breast, prostate and lung cancers. This tumor is usually diagnosed at the average age of 69 years, in which 60% are over the age of 65 and 36% are over 75 years. Although many therapeutic approaches for CRC are available today, this pathology is still responsible for a high number of deaths, representing the fourth cause of mortality due to cancer diseases (693.933 deaths, 8.5% of all tumor deaths in 2012) [1,2]. Over the years, the geographical distribution of CRC has changed, showing today a higher incidence and mortality rates mainly in the more developed countries (Australia/New Zealand/Europe) and in the less developed areas (Eastern Europe/Russia/South America) respectively [3]. Despite the epidemiological data described above, CRC mortality rate is declining in many developed countries worldwide thanks to the improvement of anti-neoplastic treatments and the new screening programs adopted [4].

Several factors, mainly related to eating habits or lifestyles, contribute to the development of colorectal cancer. Among these risk factors, a leading role is played by obesity [5], cigarette smoking [6], alcohol abuse [7] and by a diet rich in fats and red meat and low in fiber and vegetables [8]. In addition to these risk factors, there are also several physiological and pathological predisposing conditions, such as age over 50 years, family history of CRC, inflammatory bowel disease (IBD) and inherited syndromes (Lynch syndrome, Familial adenomatous polyposis, etc.) [9,10].

Beside these well-recognized risk factors, CRC onset is often associated with several genetic mutations affecting key genes involved in the regulation of cell cycle, cell proliferation and apoptosis. Among these, mutations occurring in *KRAS*, *APC*, *TP53* and *PIK3CA* genes are the most observed in CRC [11-13]. Other inactivating mutations affect genes involved in the Mismatch Repair system leading to an accumulation of somatic mutations that promote cells’ neoplastic transformation [14,15]. Finally, several studies indicate that CRC development is age-related and that aging is associated with the loss of gene regulation mechanisms that lead to a further accumulation of oncogene mutations [16,17]. Recently, aging has also been linked to epigenetic modifications capable of altering cellular homeostasis thus favoring the development of tumor pathologies [17–19].

Although colorectal cancer mortality is lower when compared to those of other cancers, the clinical management of CRC is often complicated by several associated comorbidities resulting in a negative health economics impact [20]. The main critical aspects for the management of colorectal cancer are the high rate of tumor relapses after surgery and the frequent and invasive diagnostic tests performed during the follow-up. Other issues are related to the high costs and the low compliance rates of colonoscopy [21] and the low specificity and sensitivity of the screening tests, such as the faecal occult blood test or the detection of CEA and CA 19.9 biomarkers, which often fail to early identify CRC tumor lesions [22,23].

On these bases, the discovery of new markers and the implementation of new diagnostic screening tests for CRC may be helpful to improve the effectiveness of diagnostic strategies, especially in individuals with high risk of tumor development [24].

In the last decade, several studies hypothesized that the development of cancer is associated with the deregulation of microRNAs (miRNAs), small non-coding RNA sequences that inhibit gene expression by binding to mRNAs inducing their degradation or the blocking of translation [25]. In particular, miRNAs can be detected in different biological samples, including serum, saliva and stool, and used as biomarkers with high specificity and sensitivity for the identification of pre-cancerous lesions [26–30]. However, conflicting data were generated on this matter. Such contradictory results may be caused by the difficulty in analyzing the huge amount of information available derived from the genome sequencing analysis [31].

The identification of novel biomarkers, linked to tumor development, is one of the main challenges of cancer research. Accordingly, the computational discovery of over-expressed or down-regulated miRNAs in CRC represents the first step of this process.

To our best knowledge, no previous studies have analyzed simultaneously all microRNA profiling datasets available on tissue samples from CRC patients. In order to identify miRNAs differentially expressed in CRC patients compared to healthy controls, a broad computational analysis of all microRNA profiling datasets, available on the Gene Expression Omnibus DataSets (GEO DataSets), was performed. Furthermore, the interaction between such miRNAs and the main genes altered in CRC were investigated along with their role in the modulation of molecular pathways known to be involved in cancer onset and progression.

## RESULTS

### microRNA profiling datasets selection

The analysis performed on GEO DataSets allowed to identify 114 microRNA profiling by array datasets (published until December 2017) concerning the CRC. However, most of these did not meet the inclusion and exclusion criteria described above because they were constructed with miRNA expression data obtained from tumor cell lines and not from CRC patients. Therefore, the used criteria allowed to select only 10 datasets in which the subsequent analyses were carried out. All the information about the selected datasets is reported in Table 1.

**Table 1:** Characteristics of the datasets selected for the study.

	**Series Accession**	**Normal** **n.**	**Cancer** **n.**	**Paired** **Samples**	**DefectiveMMR Samples**	**Sample Type**	**Platform**	**Author ref.**	**Identified miRNAs**		
	GSE18392	29	116	Yes	19	Fresh frozen tissue	Illumina GPL8178	Sarver, A. L. et al, 2009 [64]	miR-31, miR-135b, miR-552, miR-592, miR-503, miR-1, miR-622, miR-10b, miR-147, miR-33b, miR-143, miR-21	
	GSE108153	21	21	Yes	Not Defined	Fresh frozen tissue	Agilent GPL19730	Zeng, Z. et al, 2017	Not Reported	
	GSE30454	20	54	No	35	FFPE tissue	Illumina GPL8179	Balaguer, F. et al, 2011 [65]	miR-1238, miR-192*, miR-362-5p, miR-938, miR-622, miR-133b, miR-16-2*, miR-30a*, miR-183, miR-486-5p	
	GSE35834	23	31	Yes	Not Defined	Fresh frozen tissue	Affymetrix GPL8786	Pizzini, S. et al, 2013 [66]	miR-143, miR-145, miR-125b, miR-21, miR-17, miR-92, miR-20, miR-100, miR-183, miR-31, miR-150, miR-139-5p, miR-244, miR-10b, miR-99a, miR-182, miR-145, miR-195, miR-497	
	GSE38389	71	69	Yes	Not Defined	Fresh frozen tissue	Exiqon miRCURY LNA GPL11039	Gaedcke, J. et al, 2012 [67]	miR-135b, miR-492, miR-542-5p, miR-584, miR-483-5p, miR-144, miR-2110, miR-652*, miR-375, miR-147b, miR-148a, miR-190, miR-26a/b, miR-338-3p	
	GSE41012	15	20	Yes	Not Defined	Fresh frozen tissue	Exiqon miRCURY LNA GPL7724	Li, X. et al, 2015	Not Reported	
	GSE41655	15	33	No	Not Defined	Fresh frozen tissue	Agilent GPL11487	Shi, X. et al, 2015	Not Reported	
	GSE49246	40	40	Yes	Not Defined	FFPE tissue	Sun Yat-Sen University Cancer Center GPL17496	Zhang, J. X. et al, 2013 [68]	miR-21-5p, miR-20a-5p, miR-103a-3p, miR-106b-5p, miR-143-5p, miR-215	
	GSE68204	8	37	Yes	Not Defined	FFPE tissue	Agilent GPL10850	Millino, C. et al, 2017 [69]	miR-572, miR-939, miR-630, miR-638, miR-575, miR-374b, miR-32, miR-186, miR-30e*, miR-150, miR-155, miR-33a, miR-324-5p, miR-200b*, miR-142-3p, miR-210, miR-1260, miR-574-3p, miR-192*, miR-29b, miR-26b, miR-30c, miR-193a-3p, miR-142-5p, miR-29c, miR-7g, miR-7, miR-200a, miR-2015	
	GSE83924	20	20	Yes	Not Defined	Fresh frozen tissue	Affymetrix GPL16384	Nagy, Z. B. et al, 2016	miR-375, miR-378, miR-139-5p, miR-133a, and miR-422a, miR-503, miR-375, miR-378, miR-139-5p, miR-133a, and miR-422a	

Of these datasets, 3 were developed by Agilent (Agilent Human miRNA Microarray), 2 developed by Affymetrix (Affymetrix miRNA Array), 2 developed by Illumina (Illumina Human MicroRNA expression beadchip), 2 developed by Exiqon – Qiagen (Exiqon miRCURY LNA microRNA array v.9.2 Extended Version), and 1 datasets was a custom platform used by Sun Yat-Sen University. Overall, the bioinformatics analysis was carried out on 703 samples of which 262 normal and 441 samples of colorectal carcinoma.

### Identification of putative miRNAs involved in CRC development

**Figure 1:**
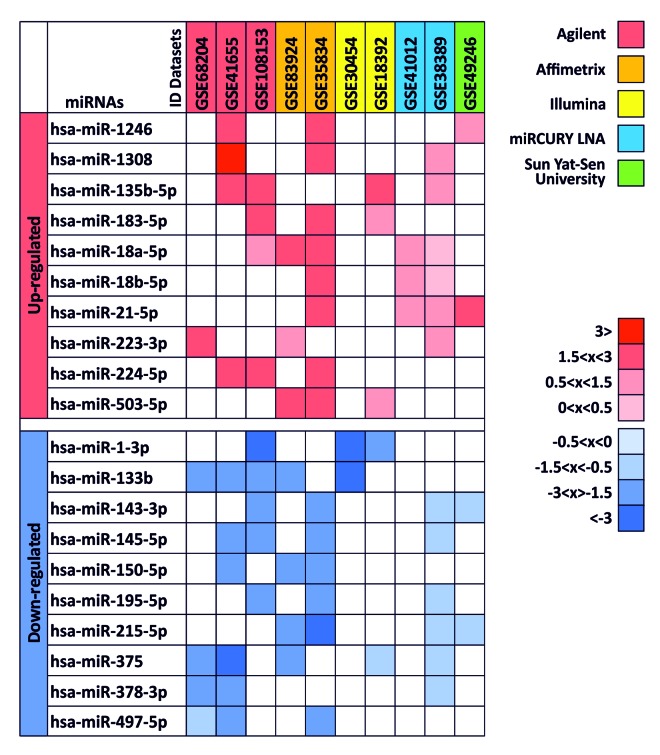
**Differentially expressed miRNAs between colorectal cancer samples and normal tissues in at least 3 of 10 datasets.** logFC values are reported with red scale boxes for up-regulated miRNAs and blue scale boxes for the down-regulated miRNAs. lgFC values were divided in “highly” (logFC ≥ 3), “moderately” (logFC 1.5 <x< 3), “lightly” (logFC 0.5 <x< 1.5) and “poorly” (logFC 0 <x< 0.5) up-regulated or down-regulated (negative logFC values). ID Datasets boxes were colored in a different manner according to the different microarray platform adopted in the dataset.

The differential analysis between colorectal cancer patients and related healthy controls or tissues revealed 20 different TOP 20 miRNAs (p < 0.01) that were differentially expressed in at least 3 of the 10 datasets analyzed (> 30% of all datasets). For each differentially expressed miRNA were reported the logFC value (Figure 1). Among these 20 miRNAs, 10 were up-regulated (hsa-miR-1246, hsa-miR-1308, hsa-miR-135b-5p, hsa-miR-183-5p, hsa-miR-18a-5p, hsa-miR-18b-5p, hsa-miR-21-5p, hsa-miR-223-3p, hsa-miR-224-5p, hsa-miR-503-5p) and 10 were down-regulated (hsa-miR-1-3p, hsa-miR-133b, hsa-miR-143-3p, hsa-miR-145-5p, hsa-miR-150-5p, hsa-miR-195-5p, hsa-miR-215-5p, hsa-miR-375, hsa-miR-378-3p and hsa-miR-497-5p). Among up-regulated miRNAs, hsa-miR-18a-5p (5 of 10 datasets) and hsa-miR-135b-5p and hsa-miR-21-5p (4 of 10 datasets) showed higher levels of expression in CRC, while among the down-regulated miRNAs remarkable were hsa-miR-375 and hsa-miR-133b (down-regulated in 5 of 10 datasets).

The up-regulated miRNA hsa-miR-1308 was excluded from the subsequent gene target and prediction pathway analyses because according to miRBase this is not a miRNA but a fragment of a tRNA.

### Gene target analysis of selected miRNAs

Gene target analysis performed by the bioinformatics tool mirDIP showed the level of interaction of the 19 computationally identified miRNAs (hsa-miR-1308 was excluded from this analysis because it is a fragment of a tRNA) with the main gene mutated or altered in CRC.

**Figure 2:**
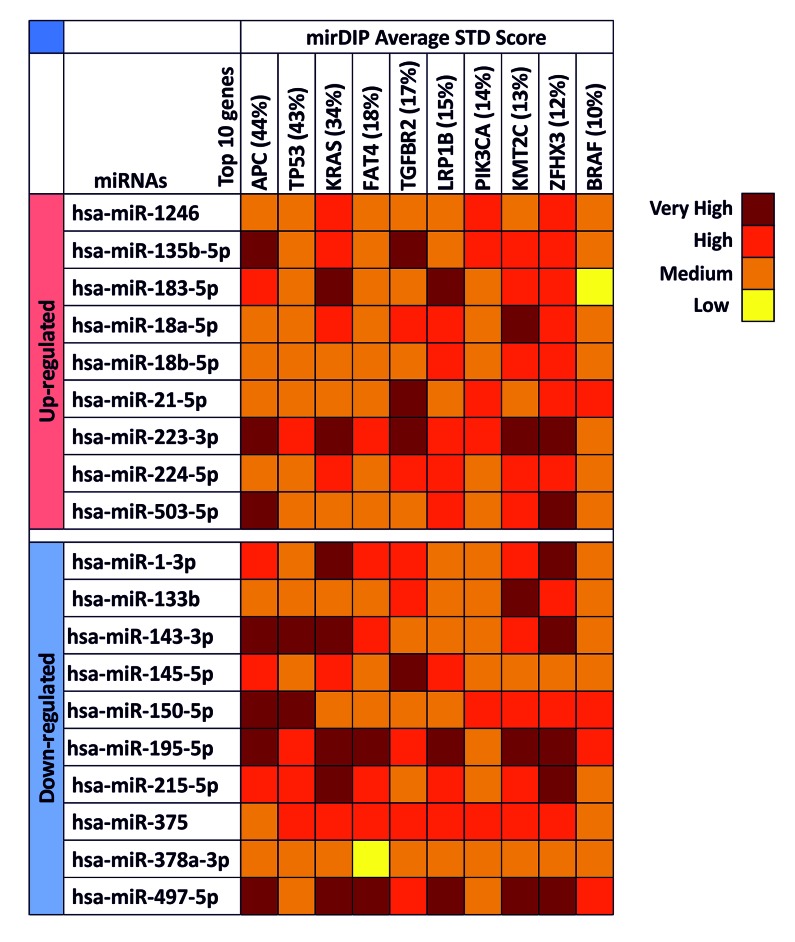
**mirDIP gene target analysis – Interaction between selected miRNAs and main altered genes in CRC.** For each miRNA is reported the level of interaction with the 10 genes involved in CRC is reported. The intensity of interaction is highlighted with a color scale ranging from dark red (very high interaction) to yellow (low interaction).

As shown in Figure 2, we take into account 10 different genes obtained from COSMIC and the mirDIP analysis revealed that all selected miRNAs were able to interact with all genes involved in CRC with high levels of specificity. In particular, for each miRNA were reported the specificity of the interaction, from low to very high specificity. Several miRNAs, such as the up-regulated hsa-miR-223-3p and the down-regulated hsa-miR-195-5p and hsa-miR-497-5p, showed very high and high interaction levels with all genes analyzed suggesting a possible role of these miRNAs in the development of colorectal cancer, while the down-regulated hsa-miR-378a-3p showed the lower level of interaction among all miRNAs. Furthermore, some genes showed to be linked with miRNAs, as in the case of *BRAF* where the only levels of high interaction are with the miRNAs up-regulated hsa-miR-21-5p and the down-regulated hsa-miR-150-5p, hsa-miR-195-5p and hsa-miR-497-5p. On the contrary, the genes linked with higher levels of specificity by miRNAs were found to be *ZHFX3* and *KRAS*, which showed in most cases high or very high levels of interaction (Figure 2).

According to this analysis, hsa-miR-223-3p, hsa-miR-195-5p, hsa-miR-497-5p are able to target and modulate both oncogene and tumor suppressor genes playing a possible key role in tumor cell development and progression.

### Two-approaches pathway prediction analysis of selected miRNAs

To understand the role of miRNAs in cancer development, the DIANA-mirPath analysis of the 19 selected highly-modulated miRNAs was performed. The two-approaches analysis was independently applied. In the first approach a prediction pathway analysis, by searching for CRC altered molecular pathways, was considered taking into account 7 different pathways (KEGG pathway), as indicated by TCGA Network. In the second approach, the same analysis, by searching for the 19 computationally selected miRNAs, was assessed.

**Figure 3:**
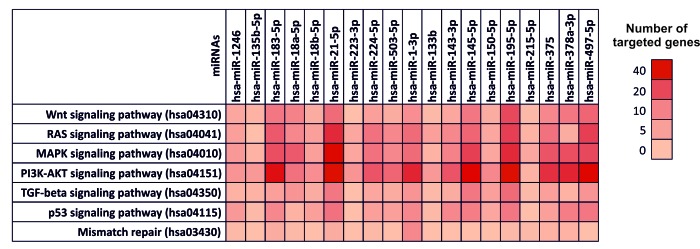
**Diana-mirPath pathway analysis – Interaction between selected miRNAs and TCGA colorectal cancer pathways.** Prediction pathway analysis of the interaction between selected miRNAs and the main genes and pathways involved in CRC development according to The Cancer Genome Atlas Network. For each miRNA is indicated the number of targeted gene within a specific pathway is indicated by highlighting the corresponding box with a color scale ranging from red (40 genes targeted) to light red (0 genes targeted).

As shown in Figure 3, almost all the selected miRNAs were able to modulate the Wnt signaling pathway (hsa04310), RAS-MAPK signaling pathways (hsa04041 and hsa04010 respectively), PI3K-AKT signaling pathway (hsa04151), TGF-β and p53 signaling pathways (hsa04350 and hsa04115 respectively) and the mismatch repair pathway (hsa03430). In particular, the most modulated pathways are the PI3K-AKT signaling pathway (hsa04151) and the MAPK signaling pathways (hsa04010), while the up-regulated miRNAs hsa-miR-183-5p and hsa-miR-21-5p and the down-regulated miRNAs hsa-miR-195-5p and hsa-miR-497-5p, showed to target the higher number of genes (214) within the 7 previously mentioned pathways (Figure 3). These miRNAs were thus able to target key genes involved in these pathways and in cancer development such as *TP53*, *APC*, several proteins of the WNT family (WNT3A,WNT5A and WNT9A) and of the MAPK family (MAPK1, MAPK8 and MAPK9), *VEGFA* and *MYC* suggesting their possible use in diagnostic and clinical practice.

On the contrary, the mismatch repair pathway (hsa03430) it was found to be the least modulated pathway from the selected miRNAs. In the same way, the miRNAs with minor gene targets were found to be hsa-miR-215-5p (11 targeted genes), hsa-miR-135b-5p (13 targeted genes), hsa-miR-223-3p (15 targeted genes) and hsa-miR-133b (16 targeted genes) (Figure 3). In Table S1 are reported the targeted gene by selected miRNAs within the aforementioned pathways (Table S1). The Figure 3 shows the number of genes targeted by the computationally selected miRNAs within several colorectal cancer related-pathway. In particular, dark red boxes indicate that those miRNAs able to target more 30-40 genes. The Table S1 contains all the genes targeted by selected miRNAs and the statistical significance (p_value) of these interactions.

**Figure 4:**
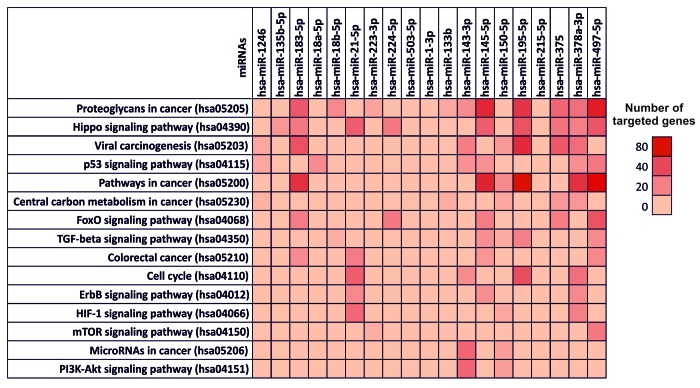
**Diana-mirPath pathway analysis – Interaction between selected miRNAs and several molecular pathways involved in cancer development.** Prediction pathway analysis of the interaction between individually selected miRNAs and the molecular and signaling pathways involved in CRC development. For each miRNA is indicated the number of targeted gene within a specific pathway by highlighting the corresponding box with a color scale ranging from red (80 genes targeted) to light red (0 genes targeted).

Subsequently, the opposite analysis was performed carrying out the second approach by entering individually the selected miRNAs. This analysis showed that the selected miRNAs were able to modulate the expression of over 1420 genes directly involved in several molecular cancer and signaling pathways, however, each gene can be targeted by several miRNAs, therefore the real number of genes regulated by the selected miRNA is 460. In Figure 4 are summarized the predicted pathways involved in cancer development and targeted by the 19 computationally selected miRNAs and their interaction with all genes of these pathways (Figure 4). All miRNAs showed to modulate the molecular pathways involved in cancer development, excluding the miRNAs hsa-miR-503-5p, hsa-miR-1-3p and hsa-miR-215-5p that have not shown interactions with any pathways. In addition, this approach confirmed the weak interaction of hsa-miR-215-5p, hsa-miR-135b-5p, hsa-miR-223-3p and hsa-miR-133b with the molecular pathways taken into account (Figure 4). Hence, this second approach confirmed the results previously obtained with the first approach. All the selected miRNAs may alter the transcriptional levels of several genes grouped in different pathways. Therefore, such miRNAs have not only a role as diagnostic markers of CRC, but could also mediate directly the processes that lead to the development colorectal cancer.

In Table S2 are reported the targeted gene by selected miRNAs within the 15 selected pathways involved in cancer development (Table S2).

## DISCUSSION

Recently, a growing body of evidence indicated the improvement of therapeutic strategies for colorectal carcinoma [32,33]. However, such improvement was not observed regarding screening and diagnostic approaches [34,35]. Conflicting data were obtained for the identification of sensitive molecular biomarkers in the context of early diagnosis [36], while promising results derived from the analysis of miRNAs.

In particular, the discovery of extracellular miRNAs, stable in several biological samples, generated a significant interest in the field of biomarker discovery and cancer treatment because the alterations of miRNAs expression levels can be used as specific and sensitive indicator for different kinds of disease, including cancer, and for the development of new personalized therapeutic strategies [37–39].

Currently, no effective biomarkers are available for the early detection of colorectal cancer. In this context, the analysis of specific miRNAs may represent a good strategy for the early diagnosis and prognosis of different tumor types, including colorectal cancer.

In previous studies it was speculated the potential role of a bioinformatics approach to identify miRNAs with high diagnostic and prognostic significance in cancer development and progression [40–44].

On these bases, in the present study a broad computational analysis taking into account all microRNA profiling by array datasets containing the miRNAs expression data of CRC biopsies and normal tissues was performed.

The analysis was conducted in 10 microRNA profiling by array datasets to identify putative miRNAs differentially expressed in CRC patients compared to healthy controls. This preliminary bioinformatics approach allowed us to identify 20 different miRNAs involved in colorectal cancer of which 10 were up-regulated and 10 down-regulated. The up-regulated miRNA hsa-miR-1308 was subsequently excluded for the gene target and pathway analyses because according to miRBase it was a fragment of a tRNA.

This preliminary analysis showed that the up-regulated hsa-miR-18a-5p and hsa-miR-21-5p and the down-regulated hsa-miR-133b and hsa-miR-375 were the most frequently found miRNAs within the 10 analyzed datasets. All these frequently modulated miRNAs in CRC are already described in literature thus highlighting the consistency and effectiveness of the performed computational analysis [45–48].

Subsequently, the gene target analysis between all the computationally identified miRNAs and the main genes mutated or altered in CRC (according to the data contained in COSMIC) was performed. Although, miRNAs are not directly involved in mutagenesis phenomena not either in reducing or favoring the onset of mutations, this analysis was important because the role of miRNAs is crucial in inhibiting over-expressed mRNAs of genes harboring activating mutations. This analysis revealed a direct and high-level interaction between selected miRNAs and several genes known to be important for CRC development and prognosis, such as *APC*, *TP53*, *KRAS* and *BRAF* [49].

In particular, the gene target analysis revealed that the miRNAs hsa-miR-223-3p, hsa-miR-195-5p and hsa-miR-497-5p showed the highest interaction levels among all selected miRNAs, with a particular specificity for the *APC*, *KRAS*, *KMT2C* and *ZFHX3*. Interestingly, the interaction values of the hsa-miR-195-5p and hsa-miR-497-5 were almost completely overlapping (9 of 10 gene interaction were the same). Of note, these 2 miRNAs were organized in a genomic cluster at the chromosome 17 in position p13.1 and this analysis suggested the same functional roles of these 2 miRNAs [50].

These 3 miRNAs were also already correlated to CRC and reported in literature. The authors described the diagnostic value of hsa-miR-223 and its role in CRC progression [51] and the importance of the miR-497∼195 cluster that were down-regulated in CRC [52–54].

Finally, the two-approaches DIANA-mirPath analysis showed that all the computational selected miRNAs have an effective role in the modulation of different colorectal cancer pathways highlighting their possible involvement in CRC development. In particular, the first approach revealed that the selected miRNAs modulate the expression levels of key genes known to be involved in the colorectal cancer pathways indicated by the TCGA Network and in the development of CRC, such as AKT family, BCL2, Cyclin family (*CCND1*, *CCND2*, *CCND3*, etc), Cyclin-dependent kinase family (*CDK4* and *CDK6*), *EGFR*, MAPK family, *TP53*, *VEGFA*, PIK3 family, etc [55–57]. In addition, the second approach confirmed the previously obtained results showing that the selected miRNAs are capable of modulating 83 different pathways, of which 15 are directly related to tumor development, interacting with more than 1400 genes. This second approach revealed a wide number of miRNAs able to target more genes compared to the first approach. The second analysis, performed by searching for a single miRNA, is a much more robust analysis that also takes into account the genes, not directly involved in the pathway, of which regulation indirectly determines a de-regulation of the analyzed pathway. Furthermore, the number of miRNAs and genes found in the second approach appears to be higher because in the second approach are also considered miRNA-gene interactions only predicted, but not yet validated. On these bases, in this study both analyses were performed to have a more comprehensive knowledge of those miRNAs able to inhibit the expression of genes involved in different cancer-related pathways. Although some of these interactions are only predicted but not yet validated, the results of this dual computational approach can provide important information on which to conduct new validation studies.

Overall, according to the gene target analysis, the analysis of the pathways showed that the up-regulated miRNAs hsa-miR-21-5p and the down-regulated miRNAs hsa-miR-195-5p and hsa-miR-497-5p are able to target the higher number of genes within the pathways reported in Table S1 and Table S2. All these miRNAs are well described in the literature and have been related to several cancer types. However, through a computational integrated analysis, for the first time was recognized a set of miRNAs strongly related to colorectal cancer gene and pathways.

Overall, these observations led us to hypothesize that such miRNAs may induce the increase of cell proliferation and inhibition of tumor cell death leading to tumor development and progression by directly inhibiting or activating (in the case of down-regulated miRNAs) key genes. Furthermore, these results represent the basis for additional studies in which define sensitivity, specificity and effectiveness of the putative miRNAs, here identified, showing highly specific interactions with genes and molecular pathways of particular interest in colorectal cancer development.

## MATERIALS AND METHODS

### Colorectal cancer microRNA profiling datasets analysis

The colorectal cancer datasets of microRNA profiling by array were selected by consulting the Gene Expression Omnibus DataSets portal (GEO DataSets) publicly available on NCBI (www.ncbi.nlm.nih.gov/geo/). In particular, the selection of the CRC datasets was carried out by entering as search terms in the advanced research tool “(("non coding rna profiling by array"[DataSet Type]) AND colorectal cancer) AND "Homo sapiens"[porgn:__txid9606]”. This preliminary research allowed to identify all CRC datasets containing miRNA expression levels information of both healthy controls and colorectal cancer patients.

This research allowed to identify several colorectal carcinoma datasets, and among these were selected only those datasets that respected the following inclusion and exclusion criteria:

Inclusion criteria, i) datasets containing the miRNA expression levels information of both cancer patients and healthy controls or containing the expression data of pathological biopsy samples and of healthy tissue counterpart; ii) datasets containing the miRNA expression data of at least 30 samples (both tumor and normal).

Exclusion criteria, i) datasets containing information only regarding cancer patients; ii) datasets containing information on the expression levels of miRNAs of tumor and normal cell lines, without considering cancer patients; iii) datasets containing information on miRNAs expression levels of serum samples; iv) datasets with obsolete miRNA annotations, datasets with no available reading keys and datasets with data not normalized and centered.

To identify the down-regulated or up-regulated miRNAs in CRC, for each selected dataset, the data matrix was downloaded to perform the differential analysis of miRNAs expression levels (fold change value) between cancer and normal samples by using GEO2R tool available on GEO DataSets. Before calculating the fold change, the miRNAs contained in all the datasets were annotated according to the last nomenclature published by miRBase (miRBase V 21) (http://www.mirbase.org/) because of the different microarray platforms taken into account [58].

The fold change values (FC) were reported as base-2 logarithm of FC (logFC) to normalize the different scales of values due to the different microarray platforms by which the datasets were constructed.

Finally, for each dataset was performed the TOP 20 list of the most statistically significant (p <0.01) up-regulated or down-regulated miRNAs was performed. This analysis allowed to identify the 10 most up-regulated and down-regulated miRNAs in colorectal cancer patients compared to healthy controls.

### Identification of putative miRNAs involved in colorectal cancer development

The previously obtained TOP 20 lists for each dataset were merged by using a bioinformatics tool, Venn Diagrams of the Bioinformatics & Evolutionary Genomics (BEG) (http://bioinformatics.psb.ugent.be/webtools/Venn/), for the comparison of sets. This approach allowed to identify only the miRNAs strongly up-regulated or down-regulated in at least 3 of the previously selected datasets. For each miRNA over-expressed or down-regulated in multiple datasets was reported the level of up-regulation and down-regulation using respectively red boxes and blue boxes. miRNAs were thus divided according to their expression levels in “highly” (logFC ≥ 3), “moderately” (logFC 1.5 <x< 3), “lightly” (logFC 0.5 <x< 1.5) and “poorly” (logFC 0 <x< 0.5) up-regulated or down-regulated (negative logFC values).

### Interaction between selected putative miRNAs and the main genes involved in colorectal cancer development and progression

By consulting the Catalogue of Somatic Mutation in Cancer (COSMIC) (http://cancer.sanger.ac.uk/cosmic) it was possible to identify the 10 most mutated genes that are known to be involved in colorectal carcinoma development and therefore have a dysregulated expression.

These genes are *APC* (44%), *TP53* (43%), *KRAS* (34%), *FAT4* (18%), *TGFBR2* (17%), *LRP1B* (15%), *PIK3CA* (14%), *KMT2C* (13%), *ZFHX3* (12%), *BRAF* (10%).

Subsequently, using the bioinformatics prediction tool microRNA Data Integration Portal (mirDIP – V 4.1.1.6, Nov 2017) (http://ophid.utoronto.ca/mirDIP) [59,60], was evaluated the interaction between the miRNA previously identified by computational analysis and the main genes mutated and altered in CRC was evaluated. For this analysis the tool mirDIP was chosen because it allows to integrate 30 different resources of human miRNA–target prediction tools thus allowing to centralize all data related to miRNAs-targets interactions.

### Interaction between selected miRNAs and main pathways in cancer

The selected miRNAs are not only involved in the alteration of the aforementioned genes, but are also involved in the regulation of a wide number of genetic targets and in the modulation of key molecular pathways of cell proliferation and cancer progression. On these bases, a pathway prediction analysis was performed to explore the implication of the selected computational miRNAs in the modulation of pathways notoriously involved in cancer development.

For this purpose, was used the bioinformatics prediction tool DIANA-mirPath (v.3) [61] was used to analyze the differentially expressed miRNA common in at least 3 of the 10 selected datasets and to predict miRNA targets in 3′-UTR gene regions according to experimentally validated miRNA interactions derived from DIANA-TarBase v7.0 algorithm [62]. These interactions (predicted and/or validated) were subsequently combined with sophisticated merging and meta-analysis algorithms by DIANA-mirPath and giving as a result the genes and pathways targeted by a specific miRNAs and the statistical significance of this interaction.

A two-approaches analysis was performed. In the first approach, the analysis of the main pathways leading to tumor development was carried out. In particular, specific pathways for colon cancer were used as search terms and analyzed, following the indications given by The Cancer Genome Atlas Network in reference to CRC [63].

To confirm this first approach, a second analysis was carried out by entering the selected computational miRNAs individually as search terms in order to identify all the genes and the pathways inhibited by them. Cancer pathways of other tumor types and the molecular pathways not directly involved in cancer development or in cell cycle and homeostasis, such as Hepatitis B pathway (hsa05161), Axon guidance pathway (hsa04360), Lysine degradation pathway (hsa00310), etc, were excluded from this analysis.

### Statistical analysis

All miRNA expression level data were already normalized by GEO2R software, therefore, no additional normalization procedures were applied to data obtained from all datasets included in this study. The p_Values of miRNAs of the gene target analysis and of the prediction pathway analysis were already calculated by mirDIP and DIANA-miPath respectively.

## Supplementary Material

Supplementary Table S1

Supplementary Table S2
