# Supplementary material for: Integrated analysis of colorectal cancer microRNA datasets: identification of microRNAs associated with tumor development
Source: Aging (Albany NY). 2018 May 18;10(5):1000–14. doi: 10.18632/aging.101444 (PMC5990389; doi:10.18632/aging.101444)
Supplement: Supplementary Table S1 [file aging-10-101444-s001.pdf]

SUPPLEMENTARY MATERIAL

Table S1. DIANA-mirPath pathway analysis - First Approach: interaction between selected miRNAs and TGCA colorectal cancer pathways.

miRNAs	p Value	N° of Targets	Gene Targets
**Wnt signaling pathway (hsa04310)**			
hsa-miR-1246	≤ 0.001	4	DVL3, TP53, PPP3CA, NFATC3
hsa-miR-135b-5p	≤ 0.001	2	APC, MYC
hsa-miR-183-5p	≤ 0.001	12	TCF7L2, ROCK2, TP53, CCND1, SMAD4, CSNK1A1, MYC, CSNK2A1, RAC1, PSEN1, NFATC3, PLCB4
hsa-miR-18a-5p	≤ 0.001	10	DAAM2, CCND2, SMAD3, JUN, CCND1, AXIN2, CSNK1A1, CSNK2A1, FOSL1, NFATC3
hsa-miR-18b-5p	≤ 0.001	3	CCND2, CCND1, CSNK1A1
hsa-miR-21-5p	≤ 0.001	14	BTRC, APC, WNT5A, CCND2, ROCK2, AXIN1, CCND1, CTNNB1, CSNK1A1, MYC, CSNK2A1, PRICKLE2, BAMBI, TBL1XR1
hsa-miR-223-3p	\	\	\
hsa-miR-224-5p	≤ 0.001	5	GSK3B, LRP6, SMAD4, MAPK8, TBL1XR1
hsa-miR-503-5p	≤ 0.001	3	CCND2, CSNK2A1, CCND3
hsa-miR-1-3p	≤ 0.001	8	CSNK2A2, CAMK2G, CTBP1, CTBP2, PLCB3, CCND1, SFRP1, DKK1
hsa-miR-133b	0.039	1	EP300
hsa-miR-143-3p	≤ 0.001	4	CSNK2A2, SENP2, FZD2, EP300
hsa-miR-145-5p	≤ 0.001	12	FZD7, GSK3B, PRKCA, WNT5A, WNT5B, PPP3CA, CCND1, CTNNB1, MYC, RAC1, WNT11, PRKACB
hsa-miR-150-5p	≤ 0.001	3	SMAD3, TP53, EP300
hsa-miR-195-5p	≤ 0.001	20	CTNNBIP1, DVL3, BTRC, CCND2, CUL1, MAPK9, CCND1, CTNNB1, MAPK8, CSNK1A1, MYC, PRKACA, EP300, WNT3A, BAMBI, NFATC3, CREBBP, TBL1XR1, WNT9A, CCND3
hsa-miR-215-5p	0.039	1	FZD5
hsa-miR-375	≤ 0.001	9	PRKCA, VANGL1, CHD8, RHOA, FZD8, FZD4, JUN, MYC, PRKX
hsa-miR-378a-3p	≤ 0.001	12	VANGL1, CCND2, ROCK2, PPP3R1, JUN, CCND1, DVL1, MYC, EP300, RBX1, NFATC3, TBL1XR1
hsa-miR-497-5p	≤ 0.001	15	DVL3, BTRC, WNT5A, CCND2, ROCK2, SMAD3, NLK, CCND1, MAPK8, CSNK1A1, MYC, CSNK2A1, TBL1XR1, WNT9A, CCND3
**RAS signaling pathway (hsa04041)**			
hsa-miR-1246	≤ 0.001	5	PIK3CB, CALM2, PTPN11, RASGRP3, RAB5B
hsa-miR-135b-5p	\	\	\
hsa-miR-183-5p	≤ 0.001	17	NRAS, STK4, IGF1R, GNB1, KRAS, PAK1, PTPN11, RAC1, FGF2, PDGFC, RALGDS, MAPK1, ABL2, GNG5, RAB5B, EFNA1, RASSF5
hsa-miR-18a-5p	≤ 0.001	9	STK4, PAK2, ETS2, GNG12, RAB5A, KIT, ARF6, RAB5C, GRIN2A

	hsa-miR-18b-5p	≤ 0.001	5	STK4, GNG12, ARF6, RAB5C, GRIN2A
	hsa-miR-21-5p	≤ 0.001	26	NFKB1, SOS2, NRAS, PAK2, GNG12, RALA, PLD1, TIAM1, IGF1R, EGFR, FASLG, MLLT4, AKT2, RASGRP1, REL, PIK3R1, TBK1, EPHA2, NF1, GAB1, PDGFD, RASGRP3, VEGFA, MAPK1, GNB4, TEK
	hsa-miR-223-3p	≤ 0.001	4	IGF1R, PIK3CD, RASGRP1, VEGFA
	hsa-miR-224-5p	≤ 0.001	13	PDGFRA, NRAS, CALM3, CALM1, PAK2, ETS1, EFNA3, IGF1R, EGFR, PIK3R3, MAPK8, CDC42, PDGFRB
	hsa-miR-503-5p	≤ 0.001	9	IGF1R, RRAS2, IKBKB, RASA1, FGF2, AKT3, HGF, VEGFA, ABL2
	hsa-miR-1-3p	≤ 0.001	14	MET, RIN1, SOS2, CALM3, CALM1, EGFR, CALM2, GNB2, AKT1, CDC42, EXOC2, PIK3CA, FGFR2, RAP1B
	hsa-miR-133b	≤ 0.001	5	MET, CALM1, IGF1R, EGFR, FGFR1
	hsa-miR-143-3p	≤ 0.001	9	CALM1, KRAS, AKT1, REL, PTPN11, HRAS, MAPK1, ABL2, RASSF5
	hsa-miR-145-5p	≤ 0.001	15	PRKCA, GNG11, NRAS, ANGPT2, EGFR, PAK1, RASA1, AKT1, RAC1, PAK4, ARF6, VEGFA, ABL2, PDGFRB, PRKACB
	hsa-miR-150-5p	≤ 0.001	4	MET, GNG11, VEGFA, FGFR1
	hsa-miR-195-5p	≤ 0.001	22	CALM1, CHUK, IGF1R, RRAS2, IKBKB, RASA1, MAPK9, GNB2, PRKACA, FGF2, CDC42, NF1, AKT3, PDGFC, PIK3CA, HGF, VEGFA, GRB2, PDGFA, EFNA1
	hsa-miR-215-5p	0.005	2	GAB1, RGL1
	hsa-miR-375	≤ 0.001	13	PRKCA, PDGFRA, FGFR3, CALM3, RAF1, IGF1R, RHOA, REL, PRKX, CDC42, PDGFC, ARF6, ABL1
	hsa-miR-378a-3p	≤ 0.001	3	CALM1, GNG12, RAB5B
	hsa-miR-497-5p	≤ 0.001	22	RIN1, SOS2, CALM1, PAK2, PIK3R2, CHUK, IGF1R, CALM2, IKBKB, AKT2, GNB2, MAPK8, FGF2, CDC42, AKT3, PIK3CA, MAP2K1, HGF, VEGFA, MAPK1, ABL2, GRB2
**MAPK signaling pathway (hsa04010)**				
	hsa-miR-1246	≤ 0.001	6	TAOK1, TP53, PPP3CA, MAP3K2, RASGRP3, NFATC3
	hsa-miR-135b-5p	0.008	2	CHUK, MYC
	hsa-miR-183-5p	≤ 0.001	19	FOS, HSPA1A, ATF2, NRAS, CRKL, STK4, ELK4, MAP3K13, KRAS, PAK1, TP53, RAPGEF2, MYC, RAC1, FGF2, MAP3K2, HSPA1B, NFATC3, MAPK1
	hsa-miR-18a-5p	≤ 0.001	17	MAPK8IP2, STK4, ELK4, PAK2, CACNB4, GNG12, MAP4K4, MAP3K1, TAOK1, JUN, HSPA8, DUSP3, STMN1, MAPK3K2, NFATC3, DUSP1, TGFBR2
	hsa-miR-18b-5p	≤ 0.001	5	STK4, ELK4, GNG12, HSPA8, STMN2
	hsa-miR-21-5p	≤ 0.001	33	NFKB1, SOS2, MAP4K2, ATF2, NRAS, CRKL, IL1B, PAK2, MAP2K7, GNG12, MAP2K3, EGFR, MAP3K4, TGFB1, FASLG, TAOK1, AKT2, DUSP10, RASGRP1, MYC, HSPA8, NF1, FAS, RPS6KA3, STMN1, MAP3K2, RASGRP3, MEF2C, MKNK2, DUSP16, MAPK1, TGFBR2, DAXX
	hsa-miR-223-3p	≤ 0.001	4	DUSP10, RASGRP1, STMN1, MEF2C
	hsa-miR-224-5p	≤ 0.001	13	PDGFRA, NRAS, PAK2, EGFR, TAB2, TAOK1, MAPK8, ZAK, CDC42, STMN1, PDGFRB, TGFB3, NR4A1

	hsa-miR-503-5p	≤ 0.001	9	DUSP2, CRKL, RRAS2, IKBKB, RASA1, FGF2, HSPA8, AKT3, CACNA2D1
	hsa-miR-1-3p	≤ 0.001	14	HSAPA1A, SOS2, MAP4K2, EGFR, BDFN, RAPGEF2, AKT1, FLNB, FLNA, CDC42, CACNA2D1, FGFR2, HSPA1B, RAP1B
	hsa-miR-133b	0.008	2	EGFR, FGFR1
	hsa-miR-143-3p	≤ 0.001	10	ELK4, MAPK7, TAB2, KRAS, AKT1, CACNA1E, HRAS, MKNK2, DUSP16, MAPK1
	hsa-miR-145-5p	≤ 0.001	22	PRKCA, NRAS, ELK4, MAPK14, MAP4K4, EGFR, TGFB1, TAOK, PAK1, MAP2K6, PPP3CA, RASA1, AKT1, MYC, RAC1, STK3, HSPA8, TGFB2, MAP3K5, PDGFRB, PRKACB, TGFB3
	hsa-miR-150-5p	≤ 0.001	4	PTPRR, TP53, FGFR1, DUSP16
	hsa-miR-195-5p	≤ 0.001	26	GNA12, ATF2, DUSP2, CRKL, CRK, MAPK14, CHUK, RRAS2, TAOK1, RPS6KA5, IKBKB, RASA1, MAPK9, MAPK8, MYC, PRKACA, FGF2, CDC42, NF1, RPS6KA3, AKT3, CACNA2D1, MKNK2, NFATC3, GRB2, PDGFA
	hsa-miR-215-5p	≤ 0.001	4	MAP3K13, TAOK1, MKNK2, HSPA1B
	hsa-miR-375	≤ 0.001	18	PRKCA, PDGFRA, DUSP6, RAF1, GADD45A, MAP3K1, TAOK1, CASP3, JUN, MYC, FLNB, CDC42, HSPA8, MAP3K8, MAP2K4, SRF
	hsa-miR-378a-3p	≤ 0.001	19	HSPA1A, CRKL, GNG12, PPP3R1, JUN, RAPGEF2, MYC, FLNA, MAPT, HSPA8, NF1, DUSP7, STMN1, HSPA1B, NFATC3, MAPK1, GRB2, RAP1B, ELK1
	hsa-miR-497-5p	≤ 0.001	20	SOS2, ATF2, CDC25B, PAK2, DUSP6, CHUK, IKBKB, AKT2, NLK, MAPK8, MYC, FGF2, CDC42, HSPA8, RPS6KA3, AKT3, MAP2K1, CACNA2D1, MAPK1, GRB2
**PI3K-AKT signaling pathway (hsa04151)**				
	hsa-miR-1246	≤ 0.001	7	PIK3CB, PPP2CA, BCL2, CDK6, TP53, CCNE1, PPP2R1B
	hsa-miR-135b-5p	≤ 0.001	6	THBS1, YWHAG, CHUK, MYC, COL4A3, PHLPP1
	hsa-miR-183-5p	≤ 0.001	31	PRLR, ITGB1, ITGB8, ATF2, NRAS, ITGA8, ATF6B, PPP2CA, YWHAG, MCL1, PCK2, HSP90AA1, ITGA5, IGF1R, GNB1, PPP2RD, PPP2R5C, KRAS, TP53, CCND1, CCNE2, MYC, RAC1, FGF2, PDGFC, EIF4E2, CREB3L2, PTEN, MAPK1, GNG5, EFNA1
	hsa-miR-18a-5p	≤ 0.001	13	IL2RG, COL27A1, CDKN1B, KRAS, RHEB, FGF11, PIK3R3, EIF4B, FOXO3, CDKN1A, SGK3, COL4A1, EFNA1
	hsa-miR-18b-5p	≤ 0.001	8	PPP2R3A, CCND2, GNG12, CCND1, ITGA2, CDKN1A, PPP2R1B, MDM2
	hsa-miR-21-5p	≤ 0.001	41	PRLR, NFKB1, SOS2, LAMB1, ATF2, NRAS, PRKAA2, THBS1, CCND2, GNG12, BCL2, IGF1R, EGFR, TRL4, CDK6, FASLG, AKT2, PTK2, BRAC1, CCND1, F2R, MYC, HSP90AB1, PIK3R1, HSP90AB1, PIK3R1, HSP90B1, EPHA2, LAMC1, COL6A3, PDGFD, FOXO3, VEGFA, PTEN, SGK3, MAPK1, PPP2R1B, COL5A2, GNB4, TEK, MDM2, COL4A1, RXRA
	hsa-miR-223-3p	≤ 0.001	7	BCL2, IGF1R, DDIT4, PIK3CD, HSP90B1, MTOR, VEGFA
	hsa-miR-224-5p	≤ 0.001	18	GSK3B, PDGFRA, NRAS, MCL1, PPP2R5D, EFNA3, IGF1R, EGFR, PIK3R3, PRKAA1, EIF4E2, FN1, PKN2, TNC, CDKN1A, PDGFRB, COL4A1, NR4A1

	hsa-miR-503-5p	≤ 0.001	15	RBL2, MCL1, CCND2, BCL2, IGF1R, IKBKB, COL6A1, CCNE2, FGF2, LAMC1, AKT3, LAMC2, HGF, VEGFA, CCND3
	hsa-miR-1-3p	≤ 0.001	27	MET, SOS2, CDK4, COL6A5, THBS1, THBS2, ITGA3, BCL2, EGFR, TLR4, YWHAQ, PPP2R5A, BRCA1, GNB2, CCND1, EIF4E, AKT1, PPP2R2A, COL1A1, HSP90B1, PIK3CA, FN1, FGFR2, ITGA6, SGK3, IL6, PPP2R1B
	hsa-miR-133b	≤ 0.001	6	MET, MCL1, EIF4EBP1, IGF1R, EGFR, FGFR1
	hsa-miR-143-3p	≤ 0.001	18	CDK4, THBS1, BCL2, YWHAB, KRAS, CDK6, IFNAR2, YWHAQ, CRTC2, AKT1, PPP2R2A, RPTOR, COL5A1, COL1A1, HRAS, CREB3L2, MAPK1, MDM2
	hsa-miR-145-5p	≤ 0.001	35	GSK3B, PRKCA, ITGB1, ITGB8, CDK4, GNG11, NRAS, THBS1, ANGPT2, ITGA5, ITGA3, EGFR, COL3A1, COL6A1, PTK2, ITGAV, ITGA11, DDIT4, CCND1, EIF4E, AKT1, MYC, COL5A1, COL1A1, HSP90AB1, RAC1, COL1A2, FN1, TNC, CDKN1A, VEGFA, COL5A2, SPP1, MDM2, PDGFRB
	hsa-miR-150-5p	≤ 0.001	9	MET, MYB, GNG11, CDKN1B, TP53, PPP2CB, EIF4E2, VEGFA, FGFR1
	hsa-miR-195-5p	≤ 0.001	41	PHLPP2, RBL2, MYB, CDK4, ATF2, IL2RB, YWHAG, MCL1, CCND1, HSP90AA1, CHUK, BCL2, YWHAB, IGF1R, PPP2R5C, CDK6, CREB1, IKBKB, GNB2, CCND1, JAK2, CCNE2, MYC, COL5A1, HSP90AB1, FGF2, PPP2CB, AKT3, PDGFC, CCNE1, PIK3CA, LAMC2, HGF, VEGFA, PPP2R1B, GRB2, SGK1, BCL2L11, CCND3, PDGFA, EFNA1
	hsa-miR-215-5p	0.026	2	HSP90AA1, BCL2L11
	hsa-miR-375	≤ 0.001	25	PRLR, PRKCA, PDGFRA, MYB, ITGB1, COL6A5, FGFR3, PRKAA2, YWHAG, MCL1, HSP90AA1, RAF1, BCL2, YWHAB, IGF1R, RPS6, MYC, HSP90AB1, YWHAZ, PPP2R3C, IRS1, PDGFC, FOXO3, MDM2, BCL2L11
	hsa-miR-378a-3p	≤ 0.001	27	PPP2R5E, MET, ITGA9, PPP2R3A, YWHAG, CCND2, HSP90AA1, GNG12, BCL2, IGF1R, TLR4, CDK6, COL3A1, IFNAR1, PIK3R3, CCND1, MYC, MLST8, PPP2R1A, PIK3R1, YWHAZ, VEGFA, PTEN, MAPK1, GNG5, GRB2, BCL2L11
	hsa-miR-497-5p	≤ 0.001	38	SOS2, CDK4, ATF2, PIK3R2, IL2RB, CCND2, HSP90AA1, CHUK, BCL2, IGF1R, RPS6, CDK6, IKBKB, COL6A1, AKT2, GNB2, CCND1, EIF4E, LPAR1, MYC, HSP90B1, FGF2, COL1A2, LAMC1, AKT3, CCNE1, PIK3CA, FN1, MAP2K1, LAMC2, HGF, COL5A3, VEGFA, MAPK1, GRB2, SGK1, MDM2, CCND3
**TGF-beta signaling pathway (hsa04350)**				
	hsa-miR-1246	≤ 0.001	2	PPP2CA, PPP2R1B
	hsa-miR-135b-5p	≤ 0.001	2	THBS1, MYC
	hsa-miR-183-5p	≤ 0.001	5	PPP2CA, ACVR2B, SMAD4, MYC, MAPK1
	hsa-miR-18a-5p	≤ 0.001	7	SMAD2, SMAD3, ACVR2A, SMAD7, PPP2R1B, TGFBR2, BMP4
	hsa-miR-18b-5p	≤ 0.001	3	SMAD2, ACVR2A, PPP2R1B
	hsa-miR-21-5p	≤ 0.001	12	THBS1, PITX2, TGFB1, ZFYVE16, MYC, SP1, BAMBI, SMAD7, MAPK1, PPP2R1B, TGFBR2, BMPR2
	hsa-miR-223-3p	\	\	\
	hsa-miR-224-5p	≤ 0.001	4	DCN, SMAD4, SMAD5, TGFB3

	hsa-miR-503-5p	≤ 0.001	3	SMURF2, LTBP1, SMAD7
	hsa-miR-1-3p	≤ 0.001	7	THBS1, ID4, E2F5, SP1, BMP7, PPP2R1B, BMPR2
	hsa-miR-133b	0.013	1	EP300
	hsa-miR-143-3p	≤ 0.001	3	THBS1, EP300, MAPK1
	hsa-miR-145-5p	≤ 0.001	12	FST, SMAD2, NODAL, THBS1, ID4, TGFB1, ACVR1, MYC, BMP2, TGFB2, BMP7, TGFB3
	hsa-miR-150-5p	≤ 0.001	5	SAMD3, BMP2, SP1, EP300, PPP2CB
	hsa-miR-195-5p	≤ 0.001	14	SMAD2, SMURF2, CUL1, MYC, RBL1, ACVR2A, SP1, EP300, PPP2CB, BAMBI, LTBP1, SMAD7, CREBBP, PPP2R1B
	hsa-miR-215-5p	0.013	1	BMP2
	hsa-miR-375	≤ 0.001	5	CDKN2B, RHOA, MYC, SMURF1, BMPR2
	hsa-miR-378a-3p	≤ 0.001	2	NODAL, SMAD7
	hsa-miR-497-5p	≤ 0.001	7	FST, SMAD3, MYC, RBL1, SP1, SMAD7, MAPK1
**p53 signaling pathway (hsa04115)**				
	hsa-miR-1246	≤ 0.001	6	BAX, CDK6, TP53, TP53I3, CCNE1, CCNG2
	hsa-miR-135b-5p	0.009	1	THBS1
	hsa-miR-183-5p	≤ 0.001	9	CCNB1, TP53, CCND1, CCNE2, EI24, SESN2, TNFRSF10B, PTEN, GTSE1
	hsa-miR-18a-5p	≤ 0.001	2	CCND1, RRM2
	hsa-miR-18b-5p	≤ 0.001	5	CCND2, CCND1, SHISA5, CDKN1A, MDM2
	hsa-miR-21-5p	≤ 0.001	13	CCNG1, THBS1, CCND2, CDK6, APAF1, CCND1, SESN1, TNFRSF10B, MDM4, FAS, SERPINEB5, PTEN, MDM2
	hsa-miR-223-3p	\	\	\
	hsa-miR-224-5p	≤ 0.001	5	IGFBP3, CDKN1A, RRM2, SERPINE1, CCNG2
	hsa-miR-503-5p	≤ 0.001	5	ZMAT3, CCND2, RRM2B, CCNE2, CCND3
	hsa-miR-1-3p	≤ 0.001	9	CDK4, THBS1, BAX, CHEK1, ATM, CCND1, SERPINB5, IGFBP3, ATR
	hsa-miR-133b	0.009	1	TNFRSF10B
	hsa-miR-143-3p	≤ 0.001	9	CDK4, THBS1, PERP, CDK6, APAF1, SESN2, BBC3, SERPINE1, MDM2
	hsa-miR-145-5p	≤ 0.001	11	CCNG1, CDK4, THBS1, ATM, CCND1, BBC3, CDKN1A, SESN3, SERPINE1, PPM1D, MDM2
	hsa-miR-150-5p	≤ 0.001	4	BAX, PERP, TP53, MDM4
	hsa-miR-195-5p	≤ 0.001	12	ZMAT3, CDK4, CCND2, RRM2B, CDK6, CCND1, CCNE1, SHISA5, SESN1, CCNE1, IGFBP3, CCND3
	hsa-miR-215-5p	\	\	\
	hsa-miR-375	≤ 0.001	5	GADD45A, CASP3, SESN1, IGFBP3, MDM2
	hsa-miR-378a-3p	≤ 0.001	11	ZMAT3, CCNB1, CCND2, CDK1, CDK6, CHEK1, CCND1, TNFRSF10B, MDM4, PTEN, SERPINE1
	hsa-miR-497-5p	≤ 0.001	12	ZMAT3, CDK4, CCND2, PERP, RRM2B, CDK6, CCND1, SHISA5, CCNE1, IGFBP3, MDM2, CCND3
**Mismatch repair (hsa03430)**				
	hsa-miR-1246	≤ 0.001	1	POLD3
	hsa-miR-135b-5p	\	\	\
	hsa-miR-183-5p	≤ 0.001	3	PCNA, MSH6, MSH2

	hsa-miR-18a-5p	≤ 0.001	1	PCNA
	hsa-miR-18b-5p	\	\	\
	hsa-miR-21-5p	≤ 0.001	3	RFC1, MSH6, MSH2
	hsa-miR-223-3p	\	\	\
	hsa-miR-224-5p	≤ 0.001	1	RPA1
	hsa-miR-503-5p	\	\	\
	hsa-miR-1-3p	≤ 0.001	9	RPA1, LIG1, POLD1, RFC2, MSH6, MSH2, RFC5, RFC3, EXO1
	hsa-miR-133b	\	\	\
	hsa-miR-143-3p	\	\	\
	hsa-miR-145-5p	\	\	\
	hsa-miR-150-5p	\	\	\
	hsa-miR-195-5p	≤ 0.001	1	RPA2
	hsa-miR-215-5p	≤ 0.001	1	MSH6
	hsa-miR-375	≤ 0.001	2	RPA1, EXO1
	hsa-miR-378a-3p	≤ 0.001	2	MLH3, EXO1
	hsa-miR-497-5p	\	\	\
