# Supplementary material for: Integrated analysis of colorectal cancer microRNA datasets: identification of microRNAs associated with tumor development
Source: Aging (Albany NY). 2018 May 18;10(5):1000–14. doi: 10.18632/aging.101444 (PMC5990389; doi:10.18632/aging.101444)
Supplement: Supplementary Table S2 [file aging-10-101444-s002.pdf]

Table S2. DIANA-mirPath pathway analysis - Second Approach: interaction between selected miRNAs and several molecular and signaling pathways involved in cancer development.

miRNAs		p Value	N° of Targets	Gene Targets
**Proteoglycans in cancer (hsa05205)**				
	hsa-miR-133b	0.0129	6	MET, PPP1CC, IGF1R, EGFR, DDX5, FGFR1
	hsa-miR-143-3p	0.0039	13	THBS1, KRAS, TFAP4, MMP2, AKT1, IGF2, FZD2, PTPN11, HRAS, MAPK1, PLAU, MDM2, CD44
	hsa-miR-145-5p	≤ 0.001	40	FZD7, ESR1, ACTB, PRKCA, STAT3, ITGB1, SMAD2, NRAS, THBS1, CAV1, WNT5A, MAPK14, ACTG1, ITGA5, IFGR1, EGFR, WNT5B, CAV2, ERBB3, TGFB1, MSN, PAK1, PTK2, ITGAV, MMP2, CCND1, CTNNB1, AKT1, MYC, MMP9, RAC1, WNT11, TGFB2, NANOG, FN1, CDKN1A, TWIST1, VEGFA, MDM2, PRKACB
	hsa-miR-183-5p	≤ 0.001	26	BRAF, ACTB, PDCD4, ITGB1, EZR, CBL, NRAS, CAV1, PPP1CC, ROCK2, RDX, IQGAP1, ITGA5, IGF1R, KRAS, PAK1, TP53, PPP1R12A, CCND1, HIF1A, MYC, ITPR1, PTPN11, RAC1, FGF2, MAPK1
	hsa-miR-18b-5p	0.0324	14	ESR1, BRAF, ACTB, STAT3, SMAD2, MAPK14, CTTN, CCND1, CTNNB1, TIMP3, ITGA2, CDKN1A, MDM2, PPP1CB
	hsa-miR-195-5p	≤ 0.001	36	ACTB, STAT3, PDCD4, SMAD2, PPP1CC, MAPK14, ACTG1, FRS2, FZD6, IGF1R, RRAS2, MSN, HSPG2, CCND1, CTNNB1, MYC, MMP9, CBLC, PRKACA, DDX5, FGF2, CDC42, AKT3, WNT3A, PIK3CA, CDKN1A, SDC4, VMP1, HGF, VEGFA, ITPR2, PLAU, GRB2, MDM2, WNT9A, CD44
	hsa-miR-223-3p	0.0179	7	STAT3, IGF1R, MMP2, PIK3CD, MMP9, MTOR, VEGFA
	hsa-miR-375	≤ 0.001	23	PRKCA, ERBB2, ITGB1, SDC1, IQGAP1, RAF1, IGF1R, RHOA, FZD8, RPS6, ANK2, FZD4, CASP3, HIF1A, MYC, FLBN, PRKX, DDX5, CDC42, TGFB2, PLCE1, CDKN1A, MDM2
	hsa-miR-378a-3p	0.0015	20	ESR1, ACTB, MET, CBL, SDC1, ARHGEF12, ROCK2, IGF1R, TLR4, MSN, PIK3R3, CCND1, MYC, FLNA, PIK3R1, VEGFA, MAPK1, GRB2, MDM2, ELK1
	hsa-miR-497-5p	≤ 0.001	43	BRAF, ACTB, PDCD4, SOS2, SMAD2, PTCH1, WNT5A, PIK3R2, PPP1CC, ACTG1, ROCK2, FRS2, FZD6, IQGAP1, IGF1R, RPS6, HSPG2, AKT2, TFAP4, MMP2, CCND1, MYC, MMP9, IGF2, ITPR1, FGF2, CDC42, AKT3, PIK3CA, FN1, CDKN1A, MAP2K1, SDC4, SMO, HGF, MTOR, VEGFA, MAPK1, GRB2, CD63, MDM2, WNT9A, CD44
**Hippo signaling pathway (hsa04390)**				
	hsa-miR-135b-5p	0.0177	10	APC, YWHAG, PPP1CC, CCND2, FZD6, BIRC5, MYC, TEAD1, LATS1, LATS2

	hsa-miR-145-5p	≤ 0.001	25	FZD7, ACTB, GSK3B, SAMD2, NF2, WNT5A, ACTG1, SNAI2, WNT5B, TGFB1, CDH1, CCND1, CTNNB1, MYC, STK3, BMP2, WNT11, TGFB2, BBC3, PARD3, SOX2, BPM7, SERPINE1, CTGF, TGFB3
	hsa-miR-183-5p	≤ 0.001	18	ACTB, YAP1, PPP2CA, TCF7L2, YWHAG, PPP1CC, MOB1B, PPP2R2D, CDH1, CCND1, SMAD4, MYC, LLGL1, FRMD6, SMAD7, LATS1, LATS2, AJUBA
	hsa-miR-195-5p	≤ 0.001	28	ACTB, DVL3, YAP1, SMAD2, BTRC, PPP2CA, YWHAG, PPP1CC, CCND2, ACTG1, GLI2, FZD6, BIRC5, YWHAB, AMOT, CDH1, CCND1, CTNNB1, MYC, PPP2R1A, PP2CB, WNT3A, SMAD7, LATS1, PPP2R1B, PARD6B, WNT9A, CCND3
	hsa-miR-21-5p	0.0065	25	ACTB, TGFBR1, YAP1, BTRC, APC, NF2, WNT5A, DLG1, CCND2, BIRC5, AXIN1, TP53BP2, TGFB1, MPP5, CCND1, CTNNB1, MYC, SAV1, TGFB2, SMAD7, LATS1, SOX2, PPP2R1B, TGFBR2, BMPR2
	hsa-miR-224-5p	0.0012	18	ACTB, GSK3B, PRKCI, DLG1, YWHAG, PPP1CC, GLI2, FZD6, MPP5, CDH1, CCND1, SMAD4, YWHAZ, LATS1, SERPINE1, PARD6B, BMPR2, TGFB3
	hsa-miR-375	≤ 0.001	19	YAP1. YWHAG, CCND2, MOB1B, YWHAB, WWC1, AMOT, FZD8, MPP5, FZD4, DLG4, CTNNA1, MYC, YWHAZ, TGFB2, PARD3, PARD6B, BMPR2, CTGF
	hsa-miR-378a-3p	0.0082	16	ACTB, ID2, YAP1, BTRC, YWHAG, CCND2, MOB1B, MPP5, CCND1, DVL1, CTNNA1, MYC, PPP2R1A, YWHAZ, SERPINE1, DVL2
	hsa-miR-497-5p	≤ 0.001	27	ACTB, DVL3, YAP1, SMAD2, BTRC, PPP2CA, WNT5A, PPP1CC, CCND2, ACTG1, GLI2, FZD6, SMAD3, BIRC5, AMOT, WWTR1, TP53BP2, CSNK1D, CCND1, MYC, FRMD6, SMAD7, LATS1, LATS2, WNT9A, CCND3, SCRIB
**Viral carcinogenesis (hsa05203)**				,
	hsa-miR-1246	≤ 0.001	6	PIK3CB, BAX, CDK6, TP53, KAT2B, CCNE1
	hsa-miR-143-3p	0.0235	17	CDK4, DLG1, HLA-E, YWHAB, KRAS, CDK6, HIST1H2BD, YWHAQ, EIF2AK2, CCR5, REL, HRAS, EP300, CREB3L2, MPAK1, LYN, MDM2
	hsa-miR-150-5p	≤ 0.001	9	BAX, PKM, CDKN1B, TP53, EGR2, KIST1H2BG, EP300, HLA-A, HIST1H2BM
	hsa-miR-183-5p	≤ 0.001	28	CDK4, ATF2, NRAS, ATF6B, YWHAG, HIST1H2BC, PKM, HIST1H4C, HLA-C, KRAS, DDX3X, CHD4, TP53, HNRNPK, EIF2AK2, CCND1, CCNE2, KAT2A, RAC1, RBPJ, GTF2H3, CREB3L2, GTF2A1, MAPK1, HLA-A, HIST1H4J, HIST1H2BO, HIST1H4E

	hsa-miR-195-5p	≤ 0.001	39	RBL2, STAT3, CDK4, ATF2, CCNA2, YWHAG, CCND2, PKM, HIST1H4C, YWHAB, TRAF5, CDK6, CHD4, CREB1, EIF2AK2, CCND1, CCNE2, UBE3A, PRKACA, DDB1, RBL1, HIST1H2BG, CDC42, EP300, CCNE1, PIK3CA, IL6ST, CDKN1A, TBP, CREBBP, TBPL1, GRB2, MDM2, CCND3, UBR4, VDAC3, HIST1H2BH, HIST1H2BE
	hsa-miR-375	0.0022	26	CCNA2, GTF2H1, YWHAG, CCND2, HIST1H4C, CDKN2B, YWHAB, RHOA, CREB3, CHD4, EGR3, EIF2AK2, CASP3, JUN, UBE3A, REL, YWHAZ, PRKX, CDC42, RBPJ, BAK1, USP7, CDKN1A, SRF, JAK1, MDM2
	hsa-miR-378a-3p	0.0201	21	YWHAG, CCND2, PKM, CDK1, CDK6, DDX3X, HIST1H2BD, CHEK1, SND1, JUN, PIK3R3, YWHAZ, HDAC2, EP300, IL6ST, MAPK1, GRB2, TRAF1, MDM2
**p53 signaling pathway (hsa04115)**				
	hsa-miR-1246	0.0437	6	BAX, CDK6, TP53, TP53I3, CCNE1, CCNG2
	hsa-miR-143-3p	0.0063	11	CDK4, THBS1, PERP, CDK6, APAF1, SESN2, TNFRSF10B, BBC3, PTEN, SERPINE1, MDM2
	hsa-miR-145-5p	0.0150	11	CCNG1, CDK4, THBS1, ATM, CCND1, SESN3, SERPINE1, PPM1D, MDM2
	hsa-miR-18a-5p	0.0023	13	CCNG1, ZMAT3, CCNB1, CDK4, CCND2, ATM, CCND1, SHISA5, CDKN1A, RRM2, PTEN, PPM1D, MDM2
	hsa-miR-378a-3p	0.0201	12	ZMAT3, CCNB1, CCND2, CDK1, CDK6, CHEK1, CCND1, TNFRSF10B, MDM4, PTEN, SERPINE1, MDM2
	hsa-miR-497-5p	≤ 0.001	18	ZMAT3, CCNB1, CDK4, CCND2, PERP, RRM2B, CDK6, CCND1, SHISA5, SESN1, TNFRSF10B, CCNE1, IGFBP3, CDKN1A, SESN3, PIDD1, MDM2, CCND3
**Pathways in cancer (hsa05200)**				
	hsa-miR-145-5p	≤ 0.001	41	FZD7, GSK3B, PRKCA, STAT3, ITGB1, CDK4, GNG11, SMAD2, NRAS, WNT5A, ITGA3, IGF1R, EGFR, WNT5B, TGFB1, TPM3, PTK2, ITGAV, CDH1, MMP2, CCND1, CTNNB1, MMP1, AKT1, MYC, MMP9, HSP90AB1, HDAC2, RAC1, WNT11, TGFB2, FN1, CDKN1A, BIRC3, STAT1, VEGFA, MDM2, PDGFRB, PRKACB, TGFB3
	hsa-miR-150-5p	0.0188	14	MET, GNG11, CXCR4, BAX, SMAD3, CDKN1B, TP53, TCEB2, BMP2, FLT3, EP300, SLC2A1, VEGFA, FGFR1
	hsa-miR-183-5p	0.0094	37	BRAF, FOS, ITGB1, CDK4, CBL, NRAS, CRKL, STK4, TCF7L2, GNA13, HSP90AA1, ROCK2, TRAF4, IGF1R, GNB1, KRAS, TPM3, TP53, CDH1, AR, CCND1, SMAD4, MSH6, CCNE2, HIF1A, MYC, MSH2, RAC1, FGF2, RALGDS, PTEN, FOXO1, MAPK1, GNG5, PLCB4, CCDC6, RASSF5

	hsa-miR-195-5p	≤ 0.001	62	DVL3, STAT3, GNA12, CDK4, CXCL8, SMAD2, CRKL, CRK, COL4A5, GNA13, HSP90AA1, GLI2, BDKRB2, FZD6, CHUK, BCL2, TRAF4, BIRC5, IGF1R, GNAI3, PTCH2, TRAF5, CDK6, PML, VHL, IKBKB, ARHGEF11, CDH1, MAPK9, GNB2, CCND1, CTNNB1, CCNE2, E2F3, MAPK8, MYC, MMP9, CBLC, PRKACA, HSP90AB1, FGF2, CDC42, EP300, GNAQ, NKX3-1, AKT3, PIAS2, WNT3A, CCNE1, PIK3CA, CDKN1A, LAMC2, HGF, VEGFA, CREBBP, GRB2, TGF, MDM2, WNT9A, XIAP, PDGFA, ADCY6
	hsa-miR-378a-3p	0.0157	37	MET, CBL, CRKL, GNAS, TGFA, ARHGEF12, GNA13, HSP90AA1, ROCK2, ARNT, GNG12, BCL2, CXCL12, IGF1R, CDK6, PML, JUN, PIK3R3, CCND1, DVL1, CTNNA1, E2F3, MYC, PIK3R1, HDAC2, EP300, RBX1, SUFU, VEGFA, PTEN, MAPK1, GNG5, GRB2, DVL2, TRAF, MDM2, GNAI1
	hsa-miR-497-5p	0.0025	56	BRAF, DVL3, SOS2, CDK4, SMAD2, PTCH1, WNT5A, PIK2R2, HSP90AA1, GLI2, ROCK2, FZD6, SMAD3, CHUK, RARB, BIRC5, IGF1R, PTCH2, CDK6, TCEB1, PML, IKBKB, AKT2, MMP2, GNB2, CCND1, LPAR1, MMP1, E2F3, MAPK8, MYC, MMP9, HSP90AB1, NOS2, FGF2, CDC42, LAMC1, AKT3, CCNE1, PIK3CA, FN1, CDKN1A, MAP2K1, SMO, LAMC2, HGF, MTOR, VEGFA, MAPK1, GRB2, MDM2, WNT9A, ADCY6
**Central carbon metabolism in cancer (hsa05230)**				
	hsa-miR-1246	0.0206	3	PIK3CB, TP53, PDK1
	hsa-miR-133b	≤ 0.001	4	MET, PKM, EGFR, FGFR1
	hsa-miR-150-5p	≤ 0.001	6	MET, PKM, TP53, FLT3, SLC2A1, FGFR1
	hsa-miR-375	0.0080	9	PDGFRA, ERBB2, FGFR3, RAF1, GLS, HK2, HIF1A, MYC, PDK1
	hsa-miR-378a-3p	0.0266	10	MET, PKM, GLS, SLC7A5, PIK3R3, MYC, PIK3R1, LDHA, PTEN, MAPK1
**FoxO signaling pathway (hsa04068)**		≤ 0.001		
	hsa-miR-145-5p	0.0331	17	IRS2, STAT3, SMAD2, BNIP3, NRAS, MAPK14, IGF1R, EGFR, TGFB1, ATM, CCND1, AKT1, IRS1, TGFB2, CDKN1A, MDM2, TGFB3
	hsa-miR-183-5p	0.0316	17	BRAF, FBXO32, CCNB1, NRAS, STK4, PCK2, IGF1R, KRAS, CCND1, SMAD4, IRS4, SOD2, PTEN, SGK3, FOXO1, MAPK1, BCL2L11
	hsa-miR-224-5p	0.0249	18	NRAS, SETD7, CDKN1B, IGF1R, EGFR, PIK3R3, CCND1, SMAD4, MAPK8, INSR, PRKAA1, MAPK12, SOD2, CDKN1A, PLK3, CCNG2, BCL2L11, TGFB3
	hsa-miR-375	0.0080	17	PRKAA2, SETD7, CCND2, RAF1, CDKN2B, IGF1R, GADD45A, HOMER2, PLK2, IRS1, TGFB2, SOD2, FOXO3, USP7, CDKN1A, MDM2, BCL2L11

	hsa-miR-497-5p	0.0025	28	BRAF, CCNB1, SOS2, SMAD2, PRKAA2, PIK3R2, CCND2, STK11, SMAD3, CHUK, IGF1R, IKBKB, AKT2, NLK, CCND1, S1PR1, MAPK8, IRS4, PRKAB2, AKT3, PIK3CA, CDKN1A, MAP2K1, MAPK1, GRB2, SGK1, MDM2, BCL2L11
**TGF-beta signaling pathway (hsa04350)**				
	hsa-miR-145-5p	0.0010	12	FST, SMAD2, NODAL, THBS1, ID4, TGFB1, ACVR1, MYC, BPM7, TGFB3
	hsa-miR-150-5p	≤ 0.001	5	SMAD3, BPM2, SP1, EP300, PPP2CB
	hsa-miR-18b-5p	0.0281	4	SMAD2, ACVR2B, ACVR2A, PPP2R1B
	hsa-miR-195-5p	0.0022	16	SMAD2, PPP2CA, SMURF2, CUL1, MYC, PPP2R1A, RBL1, ACVR2A, SP1, EP300, PPP2CB, BAMBI, LTBP1, SMAD7, CREBBP, PPP2R1B
	hsa-miR-497-5p	≤ 0.001	13	FST, SMAD2, PPP2CA, ACVR1B, SMURF2, SMAD3. ID4, ACVR2B, MYC, RBL1, SP1, SMAD7, MAPK1
**Colorectal cancer (hsa05210)**				
	hsa-miR-145-5p	0.0022	10	GSK3B, SMAD2, TGFB1, CCND1, CTNNB1, AKT1, MYC, RAC1, TGFB2, TGFB3
	hsa-miR-183-5p	0.0030	13	BRAF, FOS, TCF7L2, KRAS, TP53, CCND1, SMAD4, MSH6, MYC, MSH2, RAC1, RALGDS, MAPK1
	hsa-miR-21-5p	≤ 0.001	17	TGFBR1, APC, BCL2, BIRC5, AXIN1, APPL1, TGFB1, AKT2, CCND1, CTNNB1, MSH6, MYC, MSH2, PIK3R1, TGFB2, MAPK1, TGFBR2
	hsa-miR-497-5p	0.0194	14	BRAF, SMAD2, PIK3R2, SMAD3, BCL2, BIRC5, AKT2, CCND1, MAPK8, MYC, AKT3, PIK3CA, MAP2K1, MAPK1
**Cell cycle (hsa04110)**				
	hsa-miR-143-3p	0.0382	13	SMC1A, CDK4, DBF4, YWHAB, WEE1, CDK6, YWHAQ, TFDP2, MCM2, CDC7, EP300, MDM2, MCM3
	hsa-miR-195-5p	0.0047	29	RBL2, CDK4, SMAD2, CDC14A, CCNA2, YWHAG, CCND2, SMC3, CUL1, YWHAB, WEE1, CDK6, CCND1, CCNE2, E2F3, MYC, RBL1, TTK, CDC7, EP300, CCNE1, CDC27, CDKN1A, PRKDC, ANAPC13, CREBBP, MDM2, CCND3, CDC25A
	hsa-miR-21-5p	0.0012	25	E2F1, SMC1A, E2F2, MDM6, CCND2, BUB1, MCM4, STAG2, WEE1, CDK6, TGFB1, TFDP2, CCND1, SKP2, E2F3, MYC, RB1, HDAC2, ANAPC5, TGFB2, PRKDC, PLK1, MDM2, MCM3, CDC25A
	hsa-miR-378a-3p	0.0119	20	CCNB1, YWHAG, CCND2, MCM4, CDK1, CDK6, BUB3, CHEK1, SMC1B, CCND1, E2F5, E2F3, MYC, YWHAZ, HDAC2, EP300, RBX1, PLK1, RAD21, MDM2
**ErbB signaling pathway (hsa04012)**				
	hsa-miR-145-5p	0.0061	12	GSK3B, PRKCA, NRAS, EGFR, ERBB3, PAK1, PTK2, AKT1, MYC, PAK4, CDKN1A, ABL2

	hsa-miR-21-5p	0.0275	14	ERBB2, SOS2, NRAS, CRKL, PAK2, MAP2K7, EGFR, AKT2, PTK2, MYC, PIK3R1, GAB1, MAP2K4, MAPK1
	hsa-miR-378a-3p	0.0201	14	CBL, CRKL, MAP2K7, TGFA, JUN, PIK3R3, MYC, PIK3R1, PAK4, NRG1, MAPK1, ABL2, GRB2, ELK1
**HIF-1 signaling pathway (hsa04066)**				
	hsa-miR-150-5p	0.0296	6	CDKN1B, TCEB2, EP300, EIF4E2, SLC2A1, VEGFA
	hsa-miR-21-5p	0.0090	22	STAT3, ERBB2, NFKB1, CUL2, GAPDH, BCL2, IGF1R, EGFR, TRL4, HK2, AKT2, PDHA2, TFRC, PIK3R1, LDHA, PFKFB2, MKNK2, ALDOA, VEGFA, MAPK1, TEK, PGK1
	hsa-miR-378a-3p	0.0201	16	IFNGR2, ARNT, BCL2, IGF1R, TLR4, ENO1, PIK3R3, TFRC, PIK3R1, LDHA, EP300, RBX1, ALDOA, VEGFA, MAPK1, SERPINE1
**mTOR signaling pathway (hsa04150)**				
	hsa-miR-223-3p	0.0258	4	DDIT4, PIK3CD, MTOR, VEGFA
	hsa-miR-497-5p	≤ 0.001	18	BRAF, RRAGD, PRKAA2, PIK3R2, STK11, RPS6, RICTOR, IKBKB, AKT2, EIF4E, RRAGA, RPS6KA3, AKT3, PIK3CA, MTOR, ULK1, VEGFA, MAPK1
**MicroRNAs in cancer (hsa05206)**				
	hsa-miR-143-3p	0.0380	23	THBS1, MAPK7, BCL2, GLS, KRAS, CDK6, KIF23, PRKCE, SLC7A1, RPTOR, PIM1, PTGS2, IRS1, DNMT3A, FSCN1, HRAS, EP300, CYP1B1, PTEN, MAPK1, PLAU, MDM2, CD44
	hsa-miR-150-5p	≤ 0.001	8	MET, CDKN1B, ZEB1, TP53, MDM4, EP300, NOTCH3, VEGFA
**PI3K-Akt signaling pathway (hsa04151)**				
	hsa-miR-145-5p	0.0061	38	GSK3B, PRKCA, ITGB1, ITGB8, CDK4, GNG11, NRAS, THBS1, IFNB1, ANGPT2, ITGA5, ITGA3, EGFR, COL3A1, COL6A1, PTK2, ITGAV, ITGA11, DDIT4, CCND1, EIF4E, AKT1, MYC, COL5A1, COL1A1, HSP90AB1, IRS1, RAC1, COL1A2, FN1, TNC, CDKN1A, VEGFA, COL5A2, SPP1, MDM2, PDGFRB
	hsa-miR-497-5p	0.0308	48	SOS2, CDK4, ATF2, PRKAA2, PPP2CA, PIK3R2, IL2RB, MCL1, CCND2, HSP90AA1, STK11, CHUK, BCL2, IGF1R, RPS6, CDK6, IKBKB, COL6A1, AKT2, CRTC2, GNB2, CCND1, EIF4E, LPAR1, MYC, HSP90AB1, HSP90B1, FGF2, COL1A2, LAMC1, AKT3, CCNE1, PIK3CA, FN1, TNC, CDKN1A, MAP2K1, LAMC2, HGF, MTOR, COL5A3, VEGFA, MAPK1, GRB2, SGK1, MDM2, BCL2L11, CCND3
